# Endolithic Fungal Species Markers for Harshest Conditions in the McMurdo Dry Valleys, Antarctica

**DOI:** 10.3390/life10020013

**Published:** 2020-02-06

**Authors:** Claudia Coleine, Nuttapon Pombubpa, Laura Zucconi, Silvano Onofri, Jason E. Stajich, Laura Selbmann

**Affiliations:** 1 Department of Ecological and Biological Sciences, University of Tuscia, 01100 Viterbo, Italy; coleine@unitus.it (C.C.); zucconi@unitus.it (L.Z.); onofri@unitus.it (S.O.); 2 Department of Microbiology and Plant Pathology and Institute of Integrative Genome Biology, University of California, Riverside, CA 92521, USA; npomb001@ucr.edu; 3 Italian National Antarctic Museum (MNA), Mycological Section, 16166 Genoa, Italy

**Keywords:** Antarctica, cryptoendolithic communities, McMurdo Dry Valleys, ITS metabarcoding, fungi, marker species

## Abstract

The microbial communities that inhabit lithic niches inside sandstone in the Antarctic McMurdo Dry Valleys of life’s limits on Earth. The cryptoendolithic communities survive in these ice-free areas that have the lowest temperatures on Earth coupled with strong thermal fluctuations, extreme aridity, oligotrophy and high levels of solar and UV radiation. In this study, based on DNA metabarcoding, targeting the fungal Internal Transcribed Spacer region 1 (ITS1) and multivariate statistical analyses, we supply the first comprehensive overview onto the fungal diversity and composition of these communities sampled over a broad geographic area of the Antarctic hyper-arid cold desert. Six locations with surfaces that experience variable sun exposure were sampled to compare communities from a common area across a gradient of environmental pressure. The Operational Taxonomic Units (OTUs) identified were primarily members of the Ascomycota phylum, comprised mostly of the Lecanoromycetes and Dothideomycetes classes. The fungal species *Friedmanniomyces endolithicus*, endemic to Antarctica, was found to be a marker species to the harshest conditions occurring in the shady, south exposed rock surfaces. Analysis of community composition showed that sun exposure was an environmental property that explained community diversity and structured endolithic colonization.

## 1. Introduction

The McMurdo Dry Valleys in Antarctica cover about 4000 square km; free of snow and ice for the 30% of the surface, they represent the largest ice-free region of the continent. The landscape includes mountain ranges, nunataks, glaciers, ice-free valleys, frozen lakes, ponds, meltwater streams, arid soils and permafrost, sand dunes, and interconnected water systems. This region represents a nearly pristine environment, largely undisturbed and uncontaminated by humans, while hosts unusual microhabitats and biological communities and unique geological features and minerals. Due to the unique geological and biological characteristics, the McMurdo Dry Valleys, as a whole, are designated as an ASMA (Antarctic Specially Managed Area) to assist planning and coordination of activities to ensure the long-term protection of this unique environment and to safeguard its values for scientific research, education, and minimize environmental impacts [1]. The McMurdo Dry Valleys include five different ASPA (Antarctic Specially Protected Areas); each ASPA has its own management plan and require specific permits for entry.

The area encompasses a cold and extremely arid desert with mean annual temperature of –20 °C, always below the freezing point [2], and annual precipitation less than 100 mm water equivalent, strong winds, strict oligotrophy and strong UV irradiation. These remarkably extreme conditions make the region an important analogue for the conditions of ancient Earth and Mars and is the most investigated area as a model environment for astrobiological studies [3,4,5]. Life in these regions, where present, is mostly dominated by endolithic microbes dwelling inside rocks [6]. The low temperature and aridity are incompatible with active life on rock surfaces, and endolithic adaptation enables microbes to exploit a protected niche as the only survival [7,8,9]. Antarctic microbial cryptoendoliths are complex and self-supporting assemblages of phototrophic and heterotrophic microorganisms, including Bacteria, Cyanobacteria, Chlorophyta and both free-living and lichen-forming fungi [10,11]. They are among the most stress-resistant organisms known to date, living to the edge of their physiological adaptability [6,12]. Being well-adapted and specialized, microbes of these communities are very sensitive to any external perturbation [13,14]. This sensitivity makes them important part of the early detection and warning system for Climate Change. Antarctica is prone to the most rapid climate and has five times the mean rate of global warming in some areas over the past 50 years [15]. Since this process is likely to intensify in the future, before irreversible changes of ecosystems occur, it is critical to gain a deep understanding of Antarctic terrestrial ecosystems and to develop tools and assays to monitor future changes [16].

Recent next-generation sequencing based studies have brought new insights into the biodiversity and composition of Antarctic cryptoendolithic communities. This has helped to distinguish functional guilds of the fungal component in these communities [11,13,14,17,18], but knowledge of the factors that structure communities remains patchy.

Understanding how biodiversity varies over an extended ice-free geographic area and in response to increasing environmental pressure, individuation of key and threatened species may give tools to understand evolutionary processes in the extremes and to model how life evolves in response to rapid environmental change [19].

With this in mind, we planned this study with a focus on Fungi as they are pivotal organisms influencing the nutrient balance and functionality of these extreme and highly oligotrophic ecosystems. Fungi are important members of community participating in recycling of organic matter by facilitating nutrient liberation and uptake. The main aim was to determine the shifts of fungal community structure under different degrees of environmental pressure imposed by sun exposure since a reduced insolation means lower temperatures, lower water availability in addition to reduced incidence of photosynthetic active radiation. We also aimed to identify reliable fungal indicator taxa and determine individual taxa distribution in relation to different environmental pressure.

## 2. Materials and Methods

### 2.1. Study Area

**Figure 1:**
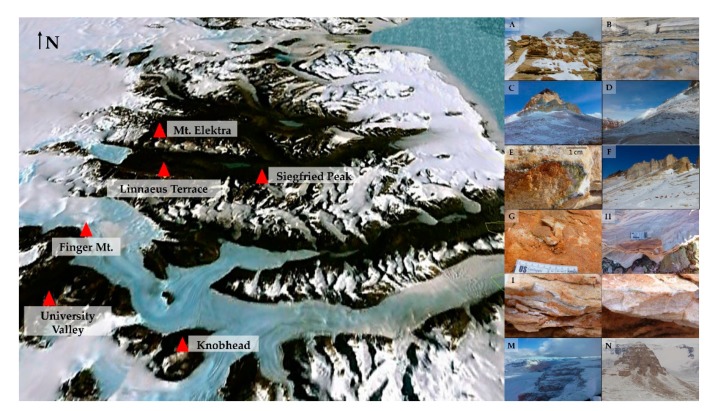
Map of the McMurdo Dry Valleys (Southern Victoria Land), by Google Earth. (**A**,**B**) Siegfried Peak north and south, respectively; (**C**,**D**) Knobhead north and south, respectively; (**E**,**F**). Sample collected at Linnaeus Terrace north and sampling site in south sun-exposure, respectively; (**G**,**H**) Samples collected at University Valley north and south, respectively; (**I**,**L**) Samples collected at Finger Mt. north and south, respectively; (**M**) Mt. Elektra north; (**N**) View of Mt. Elektra peak (southern surface has not been sampled).

Sandstones were collected in triplicate in six locations of the McMurdo Dry Valleys, Southern Victoria Land (Continental Antarctica), ranging from 1620 (Siegfried Peak) to 2150 m a.s.l. (Knobhead) during the XXXI Italian Antarctic Expedition (Dec. 2015–Jan. 2016) (Figure 1, Table 1). When possible, opposite sun-exposed surfaces were sampled to test for community composition changes in response to varied environmental condition. Rocks from Mt. Elektra south sun-exposed surface are missing because the weather conditions hampered the sampling activity. 

Sandstones were excised aseptically using a geological hammer and chisel and collected in triplicate. Samples were placed in sterile bags, preserved at −20 °C immediately upon collection to avoid contamination and transported and stored at −20 °C at the University of Tuscia (Viterbo, Italy) until processing.

### 2.2. Metabarcoding Sequencing

Rocks were crushed in sterile conditions using hammer and chisel; metagenomic DNA was extracted from 1 g of powdered rocks using MOBIO Power Soil DNA Extraction kit (MOBIO Laboratories, Carlsbad, CA, USA). ITS1F (CTTGGTCATTTAGAGGAAGTAA) and ITS2 (GCTGCGTTCTTCATCGATGC) primers were used to amplify the Internal Transcribed Spacer region 1(ITS1) for the fungal community according to Smith and Peay’s Illumina MiSeq protocol [20]. PCR reactions were performed in a total volume of 25 μL, containing 1 μL of each primer, 12.5 μL of Taq DNA Polymerase (Thermo Fischer Scientific Inc., Waltham, MA, USA), 9.5 μL of nuclease-free water (Sigma-Aldrich, St. Louis, MO, USA) and 5 ng of DNA. Briefly, PCR conditions were as above: initial denaturation at 93 °C for 3 min, 35 cycles of denaturation at 95 °C for 45 s, annealing at 50 °C for 1 min, extension at 72 °C for 90 s, followed by a final extension at 72 °C for 10 min in an automated thermal cycler (BioRad, Hercules, CA, USA). Amplicons were purified using NucleoSpin Gel and PCR Clean-up kit (Macherey-Nagel, Hoerdt, France), quantified using Qubit dsDNA HS Assay Kit (Life Technologies, Camarillo, CA, USA) and then barcoded and pooled to produce equimolar mixture. Metabarcoding sequencing (paired-end reads, 2 × 300 bp) was performed on Illumina platform at the Institute for Integrative Genome Biology, University of California, Riverside.

Three replicates for each site were extracted, amplified and sequenced; all replicates datasets have been merged to increase the amount of sequence information.

DNA concentration undetectable and PCR failure prevented sequencing the Linnaeus Terrace south samples.

Raw sequencing data have been archived in NCBI SRA database linked to BioProject accession number PRJNA453198.

### 2.3. Bioinformatics

The ITS1 amplicon sequencing dataset was processed with AMPtk: Amplicon ToolKit for NGS data (formally UFITS) (https://github.com/nextgenusfs/amptk) v.1.0.0 [21] following Coleine et al. [18]. Briefly, barcodes and primers were removed from raw data after demultiplexing. Reads were then subjected to quality trimming to a maximum of 300 bp and discarding reads less than 100 bp in length, and chimera removal utilizing USEARCH with default parameters v. 9.1.13 [22]. Sequence quality filtering was performed with the expected error parameter of 0.9 [23] and the cleaned dataset was clustered with UPARSE using a 97% percent identity parameter to generate the Operational Taxonomic Units (OTUs). Global singletons and rare taxa (<5 reads) were eliminated as likely false positives due to sequencing errors, following Lindahl et al. [24]. Finally, taxonomic identification was performed with hybrid database SINTAX/UTAX [22].

The three replicates datasets were extracted, sequenced and analyzed separately and then merged to increase the amount of sequence information.

### 2.4. Downstream Analysis

Biodiversity indices such as richness in species (S), Shannon’s diversity [25] and Simpson’s (1-D) dominance [26] indices were calculated using Primer-E v7 software (PRIMER-E Ltd. Plymouth, UK) following Selbmann et al. [13] and then were compared by two-way ANOVA (Tukey test) to test for effect of sun exposure using the statistical software SigmaStat 2.0 (Jandel Engineering Ltd., Leighton Buzzard, UK) (*p* < 0.05). 

Since changes in sequence counts can indicate relative changes in abundance [27], we have also compared per-OTU mean reads counts across the north and south sun exposure groups to calculate mean effect size with 95% confidence interval (*p* < 0.05). 

The effect of sun exposure was tested using PERMANOVA with the “adonis” function in the “vegan” package in R [28] and changes in community composition were displayed with Non-Metric Multidimensional Scaling (NMDS). Analysis was performed both on incidence (Jaccard index) and abundance (Bray–Curtis matrix) data using PAST v.2.17 software (PAleontological Statistics) [29]. Abundance reads data were square-root transformed values. Analyses were carried out with 999 permutations.

We used Indicator Species Analysis run in R (v2.14.0, R Development Core Team, 2011) using the “package” labdsv” [30] (http://ecology.msu.montana.edu/labdsv/R) and test “indval” (Dufrene and Legendre, 1997). Indicator values range from 0 to 1, with higher values for stronger indicators; only significant species at *p* = 0.05 that are predicted to be specific for north or south exposure respectively were considered good indicators.

## 3. Results 

### 3.1. Taxonomy and Biodiversity Analysis

After pre-processing, a total of 1,439,745 valid paired sequence reads were obtained, ranging from 83,413 to 260,255 reads per sample (Table S1). The further cleaned dataset, after quality trimming and filtering steps, was clustered with UPARSE using a 97% percent identity, generated 334 Operational Taxonomic Units OTUs (OTUs), resulting in a total of 251, after singletons and rare taxa (<5 reads) removal (83 out of 334 OTUs) (Table S2).

The majority of the identified fungal sequences recovered among all samples belonged to the Ascomycota, followed by Basidiomycota and Mortierellomycotina in lowest percentage; the relative abundance of these phyla did not vary across different sun-exposed locations (*p* > 0.05).

**Figure 2:**
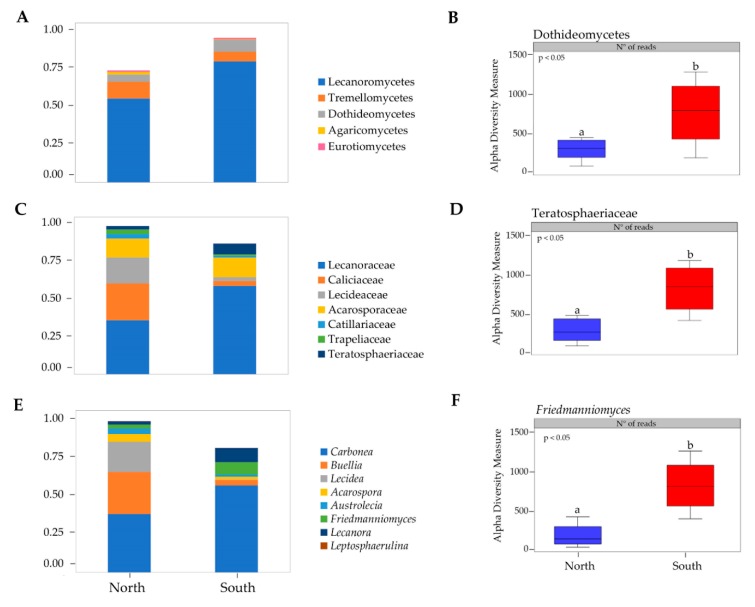
**(A**) Fungal taxonomic composition bar plot at class level; (**B**) alpha diversity measure of Dothideomycetes class; (**C**) Fungal taxonomic composition bar plot at family level; (**D**) alpha diversity measure of Teratosphaeriaceae family; (**E**) Fungal taxonomic composition bar plot at genus level; (**F**) alpha diversity measure of *Friedmanniomyces* genus. Taxa with <1% abundance were not included. Boxplots show 25th and 75th percentile, while error bars 1st and 99th. percentile. Tukey HSD significant differences (*P* < 0.05) are indicated by different letters. Classes (Leotiomycetes, Sordariomycetes, Taphrinomycetes, Basidiobolomycetes, Arthoniomycetes, Mortierellomycetes, and Pezizomycetes), families (Didymellaceae, Extremaceae, Taphrinaceae, Herpotrichiellaceae, and Cladosporiaceae) and genera (*Pleopsidium*, *Extremus*, *Taphrina*, *Elasticomyces*, *Cryomyces*, *Cladophialophora*, and *Knufia*) with <1% abundance were not showed.

Fourteen classes were observed across all samples (Table S2), where the most abundant were Lecanoromycetes, Tremellomycetes, Dothideomycetes and Eurotiomycetes (Figure 2A). Alpha diversity measure was compared for each fungal class by sun exposure and ANOVA analysis, computing abundance data, showed significant differences within Dothideomycetes (*p* < 0.05) (Figure 2B). 

Twenty-five families were identified across all sites (Table S1); Caliciaceae, Acarosporaceae, Lecideaceae, Lecanoraceae, and Teratosphaeriaceae were predominant (Figure 2C). Teratosphaeriaceae was the only fungal family with significantly higher relative abundance in the southern exposed samples (*p* < 0.05) (Figure 2D).

*Friedmanniomyces*, among the 31 fungal genera identified, was significantly more abundant in southern rocks as shown in the taxonomic composition bar plots (ANOVA, *p* < 0.05) (Figure 2E,F). 

### 3.2. Biodiversity and Community Composition Patterns

The number of reads (143,975 ± 47,529), richness (S) (92 ± 13), Shannon’ index (H’) (2 ± 0.23), and the Simpson’ index of dominance (1–D) (0.7 ± 0.07) were calculated on averaged data for each site and reported in Table S1. 

**Figure 3:**
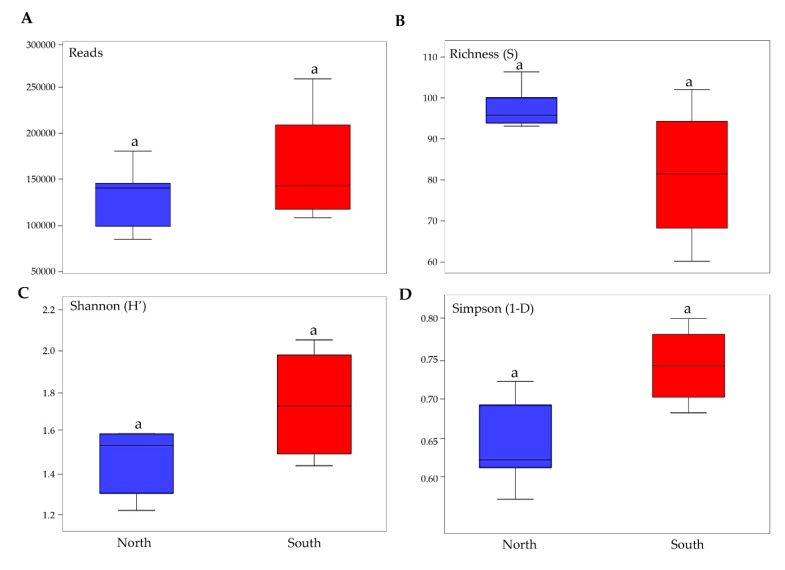
Boxplots show biodiversity measures in north and south sun-exposed cryptoendolithic communities. (**A**) Number of reads, (**B**) richness, (**C**) Shannon’s index, (**D**) Simpson’s index. Boxplots show 25th and 75th percentile, while error bars show 1st and 99th percentile. Letters indicate no significant differences in one-way ANOVA Tukey test (significant for *p* < 0.05).

Pairwise comparisons of biodiversity indices that were performed among north and south exposed rock communities indicated that relative abundance, richness in species and biodiversity did not vary amongst the two groups of exposed communities (Tuckey’ Test, *p* > 0.05) (Figure 3). 

To investigate the effect of sun exposure on fungal biodiversity and composition, a NMDS analysis was performed computing both incidence and abundance data. Overall, the NMDS plot revealed a significant influence of sun exposure on fungal communities’ composition, showing the majority of samples clustered together by sun exposure according to PERMANOVA analysis (*p* < 0.05). Few north rock samples were clustered with south rocks, in particular from Mt. Elektra north and Finger Mt. (Figure 3A).

### 3.3. Unique and Indicator Species across North and South Sun Exposure 

**Figure 4:**
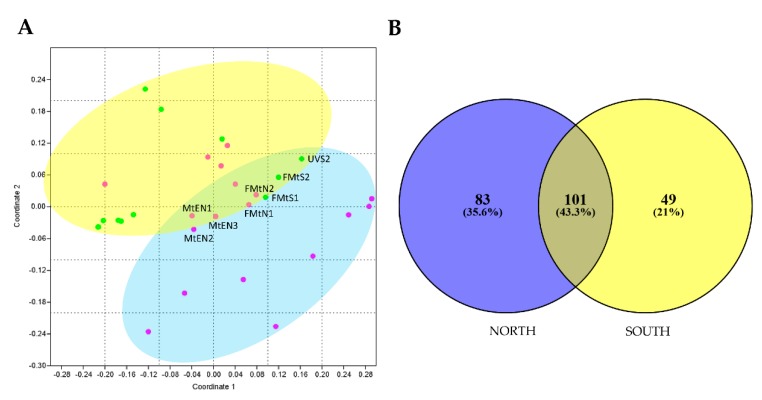
**(A**) Non-metric multidimensional scaling (NMDS) ordination plots for fungal cryptoendolithic communities differently sun-exposed, based on square-root transformed abundance data (PERMANOVA, *p* < 0.05). Stress value is 0.07. Since both approaches produced similar results, we showed results based on abundance only. MtEN1, MtEN2, MtEN3 = Mt. Elektra north sample 1, 2, and 3, respectively; FMtN1 and FMtN2 = Finger Mt. north sample 1 and 2, respectively; FMtS1 and FMtS2 = Finger Mt. south sample 1 and 2, respectively; UVS2 = University Valley south sample 2. (**B**) Venn diagram shows the distribution of fungal OTUs between north and south exposition. Both the percentages of OTUs that were shared and found exclusively in each sun exposure are indicated.

In Figure 4B, Venn diagram showed that a substantial fraction (43.3% of the total) of fungal OTUs was shared between the two groups, while 83 (35.6%) and 49 (21%) OTUs were found exclusively in north and south sun-exposed communities, respectively. In particular, most of 83 taxa unique for north exposed rocks belonged to families Acarosporaceae, Caliciaceae, Catillariaceae, and Trapeliaceae (class Lecanoromycetes); while, in southern communities were retrieved OTUs mostly belonged to Dothideomycetes (families Didymellaceae, Pleosporaceae, Teratospheriaceae such as *Meristemomyces*) and few to Eurotiomycetes (i.e., genus *Knufia* in the order Chaetothyriales).

Indicator species analysis revealed 14 and 16 indicator OTUs in north and south sun exposure, respectively. Indicator taxa from rocks from northern surfaces belonged Ascomycota which included mostly Lecanoromycetes, followed by Dothideomycetes, Sordariomycetes, Taphrinomycetes and Archaeorhizomycetes. Indicator OTUs detected from southern sites belonged to Lecanoromycetes, Dothideomycetes, Leotiomycetes, Pezizomycetes, and Archaeorhizomycetes. Two OTUs belonged to Basidiomycota (Tremellomycetes). Specifically, the genera *Archaeorhizomyces*, *Austrolecia*, *Buellia*, *Paraphaeosphaeria*, *Fusarium*, *Leptosphaerulina* (sp. *australis*), *Lecidea* (sp. *cancriformis*) and *Saitoella* (sp. *coloradoensis*) were found in northern samples, while the black yeast *Friedmanniomyces endolithicus* was found in southern samples (Table S3).

## 4. Discussion

In this study, we performed a wide survey of rocks collected in 6 locations right over the McMurdo Dry Valley, the largest ice-free area in Antarctica, sampling the main accessible sandstone outcrops and considering opposite sun-exposed surfaces to test the effect of sunlight on fungal diversity.

Using ITS1 metabarcoding, we identified Lecanoromycetes, Tremellomycetes, Dothideomycetes and Eurotiomycetes as predominant classes. The majority of the Ascomycota were identified as members of families Caliciaceae, Acarosporaceae, Lecideaceae Lecanoraceae, and Teratosphaeriaceae. Even though members of Extremaceae, Taphrinaceae, and Herpotrichiellaceae were frequently isolated in the almost last 20 years cultivation-based analysis [31,32,33,34,35], taxa belonging to these groups were almost absent in this study. Among the identified genera, species belonging to lichenized genera as *Acarospora, Lecidea,* and *Buellia* and dothideomycetous *Friedmanniomyces* spp. were the most abundant, confirming previous molecular studies [13,14].

When taxonomic composition for each fungal class was compared in respect to sun exposure, all phyla in fungal communities did not vary across northern and southern surfaces. Among the identified twenty-five families Caliciaceae, Acarosporaceae, Lecideaceae, Lecanoraceae, and Teratosphaeriaceae were predominant (Figure 2C). Teratosphaeriaceae was the only with significantly higher relative abundance in the southern exposed samples across the twenty-five identified families (*p* = 0.05). We also identified *Friedmanniomyces* as the only fungal genus that contributed significantly to endolithic sun exposure patterning as shown in Figure 2F. These results agree with previous findings reported in Coleine et al. [18], where authors found all functional groups of fungi more abundant in communities sampled in north-exposed rocks, with the exception of Rock-Inhabiting Fungi (RIF) and black fungi, that predominated in southern expositions, where conditions are much more extreme. Black fungi, indeed, are well known to be particularly adapted to highly diverse stressing environments such as saltpans, hydrocarbon-contaminated sites, exposed bare rocks and monuments, icy habitats, deserts and solar panels and building roofs [36,37,38,39,40,41]. Their extraordinary abilities to resurrect from dry conditions, e.g., [42] and to tolerate almost chemical and physical stresses including extreme pH, high and low temperature, desiccation, UV and ionizing radiation and alpha particles [43,44,45,46,47,48,49], allow these extremo-tolerant organisms to succeed when conditions are incompatible for the most [50].

Due to the harshest conditions of the study area, the diversity (Shannon’s index ~ 2) and richness (~90 per sample) of the fungal community observed in this study were relatively lower compared to other more temperate habitats [51]. Although biodiversity indices did not show significant differences across northern and southern sun-exposed communities, the effect of sun exposure was reported on community composition as revealed by NMDS/PERMANOVA analysis, showing that most samples clustered by sun exposure [18]. The effect of sunlight was also recently tested by a preliminary molecular survey, based on DGGE and qPCR techniques, of 48 rocks with north and south sun exposure, collected in Victoria Land along an altitudinal transect from 834 to 3100 m a.s.l. [52]. There, it has been found that differences in sun radiation influenced community composition and relative abundance of the three main biological compartments (fungi, algae and bacteria). In Coleine et al. [53], a first untargeted metabolomics approach, based on ultra-high-performance liquid chromatography (UHPLC) and Mass Spectrometry has been performed to give insights on the functionality of Antarctic endoliths and demonstrate how the metabolic response shifts across variation due to sun exposure, detecting altered metabolites unique for north and south, respectively.

We also identified a core mycobiome composed of 101 OTUs that were shared across two exposures, as represented by the overlapping areas of circles in Venn diagram, suggesting that these taxa may play important roles in the function of the community and be critical to the function of that type of community [54]. The primary effect of sun exposure concerned OTU presence/absence in north and south sun-exposed sites. We, indeed, found 83 taxa (35% of the total) unique for north (i.e., OTUs belonging to Acarosporaceae, Caliciaceae, Catillariaceae, and Trapeliaceae) and 49 (21%) for south (i.e., OTUs belonging to Didymellaceae, Pleosporaceae, and Teratospheriaceae).

In this study, using indicator species analysis, we were able to identify marker species that may serve as a measure of the environmental conditions that exist in a given sun exposure in McMurdo Dry Valleys. We showed that differences in sun-exposed communities were detected with indicator species, supporting hypothesis that Antarctic cryptoendolithic communities are mainly structured by sunlight. Among the identified marker species for northern samples, Lecanoromycetes predominated. Lichens are considered extraordinary well adapted to the lithic lifestyle, due to their low mineral nutrient demand, high freezing tolerance, and ability to be photosynthetically active at suboptimal temperatures [55]. Moreover, due to the large range in growth rates coupled with the simplicity of measuring lichen growth, they are regarded as an excellent tool for the detection of climate change in continental Antarctica [56].

The species *Friedmanniomyces endolithicus* [57] was, instead, found as marker species to the harshest conditions occurring in the shady, south exposed rock surfaces. This species is the most widespread and frequently isolated fungus, retrieved over 20-years of Italian Antarctic campaigns, up to 3300 m asl and 96 km of sea distance [14,34], suggesting a high degree of adaptation to the prohibitive environmental conditions of Antarctic desert. Proteomic studies have highlighted that responses to sub-optimal temperature are related to a downregulation of response rather than a heat-shock protein over-expression [58] and the ability of this fungus to tolerate acute doses of gamma radiation (up to 400 Gy) was also demonstrated [59]. Recently, the whole genome *Friedmanniomyces endolithicus* CCFEE 5311 was sequenced and assembled, resulting in 46.75 Mbp and 18,027 predicted proteins; genomic traits in response to salt, X-rays, cold and DNA damage stresses have been identified, confirming exceptional poly-heterotolerance of this species to survive across a wide variety of stresses [60].

In conclusion, in this study, based on the largest rocks survey in the McMurdo Dry Valleys, we reliably demonstrated that sun exposure has an extensive effect mainly on the fungal diversity and composition of the Antarctic cryptoendolithic communities and we were able to identify, to our knowledge, for the first time, the specific taxa that were associated with differently sun-exposed habitats. 

## Figures and Tables

**Table 1:** Characteristics of sampling sites in McMurdo Dry Valleys (Southern Victoria Land): sun-exposure, altitude and relative humidity (measured when sampling), and geographic coordinates.

Site	Sun Exposure	Altitude (m a.s.l.)	Relative Humidity (%)	Coordinates
Siegfried Peak	North	1620	52	77°34′43.3″ S 161°47′11.7″ E
Siegfried Peak	South	1620	54	77°34′39.9″ S 161°47′17.4″ E
Linnaeus Terrace	North	1649	58	77°36′01.3″ S 161°05′00.5″ E
Linnaeus Terrace	South	1761	68	77°37′09.9″ S 161°11′50.8″ E
Finger Mt.	North	1720	35	77°45′0.9″ S 160°44′44.5″ E
Finger Mt.	South	1720	35	77°45′10.3″ S 160°44′40.3″ E
Mt. Elektra	North	2080	63	77°29′28.0″ S 160°54′16.4″ E
University Valley	North	2090	18	77°52′27.6″ S 160°44′38.9″ E
University Valley	South	2200	39	77°52′21.6″ S 160°45′20.5″ E
Knobhead	North	2150	50	77°54′37.8″ S 161°34′48.8″ E
Knobhead	South	2150	38	77°54′43.6″ S 161°34′39.3″ E

## Supplementary Materials

The following are available online at https://www.mdpi.com/2075-1729/10/2/13/s1. Table S1. Diversity metrics for fungal ITS rRNA gene sequencing for each site. Table S2. Fungal OTU table with taxonomy assignment 97% of identity. Table S3. Indicator species predicted to be specific for north or south exposure respectively at *p* = 0.05.
